# The Influence of Breathing on the Central Nervous System

**DOI:** 10.7759/cureus.2724

**Published:** 2018-06-01

**Authors:** Bruno Bordoni, Shahin Purgol, Annalisa Bizzarri, Maddalena Modica, Bruno Morabito

**Affiliations:** 1 Cardiology, Foundation Don Carlo Gnocchi Irccs, Department of Cardiology, Institute of Hospitalization and Care, Milano, ITA; 2 Osteopathy, National University of Medical Sciences (usa), Naples, USA; 3 Osteopathy, CRESO, School of Osteopathic Centre for Research and Studies, Fano, ITA; 4 Department of Cardiology, Foundation Don Carlo Gnocchi Irccs, Department of Cardiology, Institute of Hospitalization and Care, Milano, ITA; 5 Osteopathy, School of Osteopathic Centre for Research and Studies, Rome, ITA

**Keywords:** diaphragm, breathing, phrenic nerve, vagus nerve, neural oscillation

## Abstract

The functions of the diaphragm do not stop locally in its anatomy but affect the whole body system. The respiratory rhythm, directly and indirectly, affects the central nervous system (CNS). This article describes and reviews these influences, containing, for the first time, information on this subject in a single text. The ability of breath to move the brain mass and determine patterns of neural oscillation will be discussed. The role of the diaphragm in influencing motor expression and its effect on intracranial blood shifts in respiratory activity will also be discussed. It is known that the diaphragm can have multiple uses in improving the symptomatological picture of chronic diseases, but there is no current, concrete data on the effects that the rehabilitative training or manual approaches could have on the patient; in particular, on his/her cognitive and cerebral aspects in general.

## Introduction and background

The diaphragm is the motor muscle of breath, which can be automatic, forced, or controlled. The diaphragm is assigned to multiple functions, both indirectly and directly, which go beyond breathing. It also promotes expectoration, vomiting, defecation, urination, swallowing, and phonation. The diaphragm influences the body metabolic balance [1-2] and stimulates the venous and lymphatic return, thereby creating the correct relationship between the stomach and the esophagus to prevent gastroesophageal reflux [3]. It is essential for correct posture and locomotion, as well as for the movement of the upper limbs [3-5]. The diaphragmatic muscle influences the emotional and psychological spheres. Inspiratory apnea is able to raise the somatic pain threshold, decreasing the painful perception [6-8].

The functions of the diaphragm do not stop locally in its anatomy but affect the whole body system. It can be called systemic breath [9]. The main nerves for the peripheral innervation of the diaphragm are the phrenic and vagus (the latter for the crural area). The phrenic nerve receives impulses from the groups of medullary neurons of the preBötzinger complex and from the neurons of the retrotrapezoid parafacial complex, which, in turn, receive higher orders from the retroambiguus nucleus of the bulb, even if the mechanisms underlying these links are not completely clarified [9]. The phrenic nerve (C3-C5) is a mixed nerve, capable of sending efferences and receiving sensitive afferences. It sends motor information to the diaphragm and senses information from the vena cava, the pericardium, the pleurae, the Glisson capsule, and the subdiaphragmatic peritoneal area [9]. The right phrenic nerve is more vertical, with less length, and possesses faster electrical conduction. In its path, the phrenic nerve performs many anastomoses and in different percentages, depending on the variability of the presence of the accessory phrenic nerves: vagus nerve, nerve subclavius, ansa cervicalis, stellate ganglion, cranial nerves XII and XI, supraclavicular nerve, and sternohyoid nerve [9]. In the subdiaphragmatic portion, the phrenic nerve continues its course. On the right, it forms one or more phrenic ganglia, which are connected to the celiac ganglion and the suprarenal gland, and, in some people, also with the sympathetic superior mesenteric ganglion; on the left, it forms a phrenic ganglion, which could connect to the sympathetic ganglia and to the adrenal gland, according to anatomical subjectivity [10]. In these phrenic ganglia, we find neuronal bodies that are sympathetic, and there is evidence that a retrograde system of information from the sympathetic ganglia trips back to the phrenic nerve, influencing diaphragmatic behavior [10].

The vagus nerve (X cranial nerve) is the longest of the cranial nerves. The vagus nerve is mixed, with motor skills (20% of efferent fibers) and sensitive (80% of afferent fibers) [11]. The vagus originates from the ambiguus nucleus, from the solitary nucleus and from the dorsal motor nucleus of the encephalic trunk, immediately caudal to the glossopharyngeal one; the dorsal nucleus (or cardiopneumoenteric nucleus) that is found in the bulb under the fourth ventricle floor gives rise to the parasympathetic preganglia fibers of the vagus coming out of the encephalic trunk [12]. These fibers reach the parasympathetic ganglia of the different viscera present in the mediastinum and in the abdomen. The nerve exiting its nuclei moves horizontally forward and oblique, to reach the jugular foramen; at this level, it crosses the bony canal, forming two ganglia [13]. The first ganglion in the jugular foramen of the vagus is the nodose or superior with sensory tasks, while the second, called jugular or inferior, has somatic relevance for the sensitivity of the auricle skin [14]. The vagus performs different anastomoses, including the sympathetic system at the cervical and abdominal levels and the phrenic nerve, the vagus nerve itself (loop or anastomosis of Galenum), the nerve XI (Lobstein anastomosis), nerve IX, and the ansa cervicalis [15-17]. In the respiration mechanism, a third nerve takes over, the hypoglossal (XII), particularly in the preinspiratory phase. This nerve is essential for the compliance of the respiratory airways, activating before the air enters the lungs: during the inhalation when the tongue is retruded [18-19]. The respiratory rhythm, directly and indirectly, affects the central nervous system (CNS).

This article describes and reviews these influences, containing, for the first time in the authors’ knowledge, information on this subject in a single text. The clinical intent is to remember that the diaphragm can have multiple utilities to improve the symptomatic picture of chronic diseases. In chronic diseases, a decline in cognitive activity takes place concomitantly with an alteration of the respiratory function observed in Chronic Obstructive Pulmonary Disease (COPD), obstructive sleep apnea (OSA), fibromyalgia, chronic heart failure (CHF), and chronic low back pain (CLPB) [20-27]. The ability of the breath to move the brain mass and determine patterns of neural oscillation will be discussed.

## Review

Breath and movement of the brain mass

Research has shown that there are forces capable of craniocaudally moving the brain mass and affecting the synthesis of cephalic-rachid fluid (CRF) [28]. Using magnetic resonance imaging (MRI), it has been shown that during systole, brain mass and the medulla oblongata move caudally and medially (23 millimeters), while in concomitance with the diastole, there is a cranial return [29]. The intervention of the respiratory diaphragm muscle is able to move the brain mass and influence the movement of the CRF, as well as increasing its production, in particular with forced breaths. During the inhalation, there is a cranial return of the central nervous system while with the exhalation, there is a movement in the caudal direction [30]. The difference between the heart and the diaphragm is that the myocardium moves the CRF faster while the diaphragm moves a larger quantity of fluid [31]. During inhalation, thoracic pressure is reduced, which affects the subarachnoid space through the venous plexus that surrounds the thoracic spine and inside the spinal canal; the decreased thoracic pressure influences the hydrostatic pressure that helps in low venous resistance and paravenous and CRF drainage [32]. The CRF protects the functions of the central nervous system (CNS), bringing nutrients, collecting metabolic cellular wastes, and regulating cerebral pressure. It is renewed three to five times a day, consisting essentially of molecules derived from blood for about 80% and the remaining from molecules produced by the brain and intrathecally [33].

There is some evidence suggesting a reduction in the amount of CRF in motion, if there is a resistance in the respiratory tract or apnea, with a decrease in the subarachnoid space [32,34]. Currently, it is not known what happens with physiotherapic, osteopathic, or manual treatments of the dysfunctional inspiratory muscle and the effect they have on CRF and improving brain function. One formulated hypothesis is that there is a relationship between a decline in chronic diaphragmatic function and cognitive function, disturbing the function and mobility of CRF. Not only does the reduction of oxygen caused by the diaphragmatic dysfunction affect the patient's cognitive function, but it is also probably due to a slowing down of the fluid. Another relationship between the diaphragm and the fluid is cough. Coughing helps expectoration and facilitates boosting fluid towards the cranial vector, stimulating the exchange of systemic immune information [35].

Further studies are needed to verify if a diaphragm-targeted training could improve the metabolism and immune response of the central nervous system. Another motivation of the response of movement of the cerebral mass and of the spinal cord during the act of breathing could be related to the creation of mechanical tension on the nervous structures, central (cranial) nerves, and peripheral nerves. The peripheral and central nervous structure is subjected to a daily mechanical stress load, as when an articulation moves, it undergoes compression and stretching. The physiological stress load allows the nerve to regenerate itself, through autocrine and paracrine substances, which are generated by the same nervous structure [36]. The breath moving the central and peripheral nervous structure would induce mechanical stress on the same structures, which stress would lead to the mechanotransduction phenomenon, maintaining the function and shape of the nervous tissue constantly. The movement generated would allow the form and function to persist; minor or altered movement would mean minor and impaired function and form. It is known that this happens with the heartbeat. For example, the head of the optic nerve moves synchronously with the cardiac cycle, with a pulsatile forward displacement during systole and an inward movement with diastole, for a maximum of 8.7 μm and a minimum of 2.9 μm [37]. The lamina cribrosa, the continuation of the contour of the sclera, moves in the opposite direction in systole, creating a stretching in the optic nerve. The constant stretching during each heartbeat creates conditions for the synthesis of some substances, such as endothelin-1 and nitric oxide synthase (NOS), to improve vascular supply to the optic nerve [38]. There are no studies on the relationship between diaphragmatic breathing and the movement of the cranial and peripheral nerves. However, we can hypothesize a stretching function similar to the heartbeat because we know that both the heart cycle and the respiratory rhythm move the brain mass and the medulla. There are no studies to check whether a specific work on rehabilitative breathing can increase the function of the central and peripheral nervous systems.

Breathing and oscillation of the neural network

The breath modulates the limbic oscillations, the cognitive and motor functions of the cortex. This process occurs with greater force when inhalation takes place through the nose; on the other hand, the effect is less forceful if the breath is carried out with an open mouth [39]. The olfactory bulb and the piriformis cortex oscillate during the breath, probably coordinating the cortical neural network linked to learning, memory, and behavior [39]. The olfactory system is connected to the limbic system and to the hippocampus (through projections of the entorhinal cortex): the type of respiratory rhythm creates specific neural excitations (depth of breath, number of breaths, speed of breath), which create specular oscillatory rhythms that propagate in different brain areas, not necessarily related to smell. These oscillations are delta (low frequency), theta (4-12 Hz), beta (they are found with odors, 30 Hz), and gamma (40-150 Hz) [39-41].

The same respiratory rhythm is recorded differently from specific brain areas, from which the neural oscillations, which allow communication between them, start. The greater the oscillations are coordinated, the greater the function expressed by the different cerebral areas involved. The diaphragm is the "diapason" of the neural system. Breathing, in particular, affects the gamma waves, which involve the neocortex (frontal, parietal, and temporal area); these areas are activated for cognitive function: memory, attention, sensory perception, problem-solving, and language processes [40]. Neural oscillations are measured in local fields potentials (LFPs) or via an electroencephalogram (EEGs), influencing the action potential or spikes of neurons [42]. Oscillations organize the spikes of neurons over time (more precise and durable synaptic connections), implementing their ability to function and communicate with different brain areas. Neural oscillations do not depend on the extent of oxygenated blood in the brain [42]. Gamma waves also influence the limbic and motor areas of the cortex [40]. The same diaphragm muscle could directly influence the neural oscillations (particularly, the delta and theta waves), through the proprioceptive and interoceptive information that its movement transmits, activating the somatosensory and insular cortex, passing through the spinal pathways [40,42]. The direct stimulation of the diaphragm, particularly when the theta waves are activated, always stimulates the cognitive activity [43]. The diaphragm stimulates limbic rhythmogenesis involving a large number of cells that are depolarized synchronously, starting from the sensory medullary pathways (proprioception and interoception) [44]. With this mechanism, we can more easily memorize the gestures (thanks to the relationship with the hippocampus) and the emotional memory [44-45]. Not only from the nose does the rhythmogenesis stimulation start with the inhalation but also from the receptorial stimulations of the muscular structure of the diaphragm.

Another structure that contributes to the creation of neural excitatory patterns connected to the breath is the pre-Bötzinger cellular complex. The latter is the ventral portion of the medulla oblongata, an important region for the respiratory rhythm, particularly for the inspiratory phase [46]. Approximately 10%-20% of the neural cells composing this complex send autonomous action potentials (10-20 mV, 0.3-0.8 s). This sending of electrical excitation takes the name of neural pacemakers [46-47]. The retrotrapezoid nucleus and the respiratory parafacial group positioned rostrally to the pre-Bötzinger group oscillate during the active expiratory phase [48]. The mechanisms underlying these fluctuations are not fully understood. The pre-Bötzinger group is linked to the hypothalamus, the amygdala, the thalamus, the cortex, and the gray periaqueductal area [49-50]. We could hypothesize that another oscillatory and synchronized means of breathing communication can also start from these areas of the medulla oblongata influencing the cognitive and emotional aspects.

Motor coordination and diaphragm

Studies on a human model have shown that the breath produces a bilateral activation of the cortex, particularly the primary motor cortex (M1), premotor cortex, and additional motor areas [50]. Cortical activations send afferents to the medullary respiratory areas (corticospinal pathways) so that the movements produced by the respiratory musculature have a sufficient quantity of oxygen. There is a bi-univocal relation between the breathing and activation of the skeletal musculature. The contraction of the diaphragm excites the respiratory areas of the M1 cortex, in which areas of activation of the musculature of the limbs are present. Probably, this proximity allows the muscles of the limbs to be activated with greater emphasis, which has the repercussions of better motor performance (coordination and strength) [42]. A deep breath is able to express a force and perform motor coordination of the musculature of the major hand (about 10% more), as compared to a non-deep or forced breath. Some chronic diseases that negatively affect the diaphragm present an impaired motor coordination, as in patients with COPD and CHF [3], as it happens in some neurological diseases, such as Parkinsons and dystonia. Further studies are needed to exhaustively determine the underlying neural processes.

Action potentials, blood volume, and breath

The diaphragm muscle has great impacts on blood, arterial, and venous circulation, influencing intracranial pressure [9]. It has been demonstrated on a human model that a variation of cerebral blood flow is able to produce action potentials, which can be recorded with EEG. These electrical responses are not exclusively attributable to the activity of glial cells or cortical neurons but to variations in intracranial pressure. An explanation could be related to the sensitivity of intracranial or endothelial epithelial layers, particularly in the areas of the blood barrier encephalic (BBE). These epithelium layers have transmural electrical potentials, which could be stimulated by pressure changes, creating adjustable electrical responses, probably passive ion-transfer mechanisms, such as sodium and potassium, between cell membranes. Blood pressure changes may directly stimulate an electrical response of brain neurons, with small variations in microvolts (0.5 Hz). During cognitive tasks, it is possible that breathing may affect intracranial pressures and create low-voltage electrical responses.

## Conclusions

In its contractions, the diaphragm muscle has systemic functional reflexes that are not only related to changes in tissue oxygen. In this article, we reviewed some functions not yet well explored, such as the neural oscillations, the movement of the brain mass, the influences that the breath has on motor activities, and the electrical responses of the brain at low voltage (the latter through variations of blood intracranial pressures). The diaphragm still has many mysteries to be unveiled, not only on the functions it exerts in the body system but also on the usefulness that a manual approach can have on the patient. Resuming the work of Morgado-Valle (in the bibliography), we can conclude with this reflection: Breath has patterns. Schemes create behavior. Breath is a behavior. Behavior represents the person. Breath reveals the person.
